# Vertical saccadic palsy and foveal retinal thinning in Niemann-Pick disease type C

**DOI:** 10.1371/journal.pone.0252825

**Published:** 2021-06-04

**Authors:** Susanne Hopf, Julia B. Hennermann, Alexander K. Schuster, Norbert Pfeiffer, Susanne Pitz

**Affiliations:** 1 Department of Ophthalmology, University Medical Center Mainz, Mainz, Germany; 2 Department of Pediatric and Adolescent Medicine, University Medical Center Mainz, Mainz, Germany; 3 Orbital Center, Ophthalmic Clinic, Bürgerhospital Frankfurt, Frankfurt, Germany; Cairo University Kasr Alainy Faculty of Medicine, EGYPT

## Abstract

**Introduction:**

Niemann-Pick type C (NPC) is a lysosomal storage disease that is progressive and life-limiting, with an estimated incidence of 1:120,000 live births. In addition to systemic manifestation with (hepato-)splenomegaly, there are a number of neurological manifestations (ataxia, dysarthria, dementia, cataplexy, epileptic seizures, and psychiatric disorders). Characteristic is vertical supranuclear gaze palsy, which is often overlooked. Early diagnosis and start of therapy improve quality of life. This study aimed to characterize oculomotor dysfunction of NPC patients, and to provide ophthalmologic data including retinal imaging.

**Methods:**

Eighteen patients with biochemically or genetically diagnosed NPC completed oculomotor and ophthalmologic examination. Ten of them performed saccadometry by infrared based video-oculography. Saccadic parameters were compared to 100 healthy controls, and were correlated with clinical variables. Another subgroup of eight patients received optical coherence tomography (OCT) of the optic disc and the macula, of which the segmented layers were analysed using a crude linear mixed model, and one adjusted for age, sex, and spherical equivalent.

**Results:**

Saccadometry revealed slowed peak velocity compared to controls most evident vertically. Peak velocity correlated negatively with SARA-Score, but correlation with clinical assessment of saccades was not significant. Clinical features in the assessment of vertical saccades were intensive blinking and head movements to initiate gaze changes, and lateral trajectory of the eyes. Macular OCT revealed significant total retinal thinning in the fovea, specifically of the outer nuclear layer and outer retinal layer. Para- and perifoveal retinal thicknesses, as well as peripapillary retinal nerve fibre layer were normal.

**Conclusions:**

Foveal thinning was revealed in NPC. It remains to be shown, whether OCT will prove to be useful to monitor progression. Saccadic impairment reflects CNS involvement and therefore is a parameter to demonstrate the progression of NPC, and potentially also the efficacy of new therapies. Saccadometry, in contrast to clinical investigation, allows the precise evaluation of saccades.

## Introduction

Niemann-Pick diseases are progressive autosomal recessive lysosomal lipid storage disorders. Niemann-Pick type A and B are defined as acid sphingomyelinase deficiencies (SMPD1 mutations) [1]. Niemann-Pick type C (NPC) is associated with defects in the endosomal-lysosomal trafficking, resulting in the accumulation of multiple lipids in the lysosomes [2]. The underlying mutations NPC1 are involved in 95% (with about 700 variants), and NPC2 mutations in the remainder [1]. The estimated incidence of NPC is 1 case per 120,000 live births [1, 2].

NPC is a neuro-visceral disease with a broad clinical spectrum, ranging from neonatal fatal manifestation to an adult onset chronic neurodegenerative disease. The visceral manifestation is mainly (hepato-)splenomegaly, the characteristic neurological signs are vertical supranuclear gaze palsy (VSGP), cataplexy, ataxia, dysarthria and progressive cognitive impairment [1, 3]. The age of onset of first neurologic symptoms determines progression and life expectancy [1, 3, 4]. The lifespan of the majority of patients ranges between 10 and 40 years of age with a proportion of 47% under 18 years of age [1, 3]. VSGP is present in 68%, however the frequency may be underestimated as often being overlooked or not recognized if not specifically investigated [3, 5]. In general, the delay between the first clinical symptom until confirmed diagnosis ranges from 2 to 6 years, and is depending on the age of onset [3]. Making the diagnosis promptly and setting up an adequate management improves quality of life [2]. The only approved treatment for NPC is miglustat, an inhibitor of glucosylceramide synthase. NPC patients on miglustat therapy remain stable or improve by approx. 70%, depending on age at neurologic manifestation [3, 5, 6]. Additionally, miglustat treatment has been reported to be associated with a reduction in risk of mortality [4].

The most common forms are late infantile NPC (onset at 2–6 years) and juvenile NPC (onset at 6–15 years) [1, 5]; both are defined by their neurodegenerative manifestation. Early infantile NPC (onset at age < 2 years) with visceral-neurodegenerative manifestation, and adult NPC (onset at age >15 years) with psychiatric-neurodegenerative manifestation occur less frequently [2]. Diagnosis can be confirmed by mutation analysis (mandatory), oxysterol analysis, chitotriosidase activity and eventually filipin test [2]. A clinical hallmark is vertical saccadic gaze palsy, while horizontal saccadic eye movements are less impaired and therefore represent a robust objective measure. It is a biomarker of diseases severity, correlating with indices of damage of brain structures, specifically with measured volumes of the corpus callosum [7], cerebellum [8], pontine area, and parietal eye field volume [2]. Reflexive saccades are generated in the parietal eye fields (PEF), while voluntary saccades are generated in the frontal eye fields (FEF); both control the rostral interstitial nuclei of the medial longitudinal fascicle (riMLF) via the superior colliculi [9].

The neuro-ophthalmologists’ role is to diagnose VSGP, and to assess response to therapy (changes in saccadic eye movement velocity) [2]. According to Pineda et al., eye movements in NPC are categorized as either “normal”, “slow ocular pursuit”, “vertical ophthalmoplegia” or “complete ophthalmoplegia” [2, 10]. Clinical investigation of eye movements however is only qualitative in nature. Thus, quantitative measures are needed for adequate disease monitoring.

Therefore, the first aim of the present study was to obtain a detailed characterization and quantitative measurement of VSGPs. This study especially focusses on oculomotor dysfunction which we support by neuro-ophthalmological examination including video-oculography. Our second aim was to report general ocular findings of 18 patients with genetically or biochemically confirmed NPC, with special interest in possible retinal alterations including quantitative retinal layer measurements by optical coherence tomography.

## Methods

This monocentric cohort study was conducted in the University Medical Center Mainz in Germany. The study was approved by the Medical Ethical Committee of the State Chamber of Medicine of Rhineland Palatinate in Mainz, Germany (reference number 837.373.14 (9612)). The patients or their parents/guardians gave written consent to perform the study and to publication of their anonymized clinical data.

### Characteristics of participants

Inclusion criteria for participants were a biochemically and/or genetically diagnosed Niemann-Pick type C disease and the cognitive ability to perform the investigations. Children under 6 years were excluded, because cooperation was regarded not to be sufficient for the examinations at that age. Eighteen NPC patients were included in this study, of whom saccadometry was performed in 10 (for logistic and time reasons of the testing). A visual acuity of better than 1.3 logMAR at far distance was required to perform saccadometric measurements. A cohort of 100 healthy individuals at a stratified age distribution served as control. Their saccadometric and demographic data were published elsewhere [11].

### Ophthalmologic and neuro-ophthalmologic examinations

The patients underwent a standardized ophthalmologic and neuro-ophthalmologic examination. Ophthalmologic examination included: non-cycloplegic auto-refraction measurements (NIDEK AR-360A, Nidek Co., Japan), monocular best corrected visual acuity (optotypes: numbers or letters) or in case of restricted compliance preferential looking test (Teller Acuity Cards binocularly), slit lamp biomicroscopy of the anterior segments, and fundoscopy of the posterior segments.

Imaging of the optic nerve head and the macula was carried out using Spectral Domain (SD) Optical Coherence Tomography (OCT) (Spectralis, Heidelberg Engineering GmbH, Heidelberg, Germany) with automatic real-time function for image averaging. We acquired a peripapillary OCT (pOCT) and a macular OCT (mOCT). The peripapillary retinal nerve fibre layer (pRNFL) was imaged with a diameter of 12° (corresponding to 3.47mm in the standard eye), and a standard corneal curvature of 7.7mm. For the mOCT, 49 horizontal single scans were obtained. After semi-automated segmentation of the retinal layers as provided by the OCT software (Heidelberg Eye Explorer version 1.10.2.0, viewing module 6.9.5.0; HEYEX, Heidelberg, Germany), all scans were assessed regarding their quality by a board-certified ophthalmologist (SH). Those with segmentation errors were corrected, or excluded in cases of poor image quality. In cases of poor data in only one sector, this sector was excluded prior analysis; if more sectors were affected, then the complete OCT dataset of this eye was excluded.

Mean retinal thickness of 9 macular layers was used for the analysis: Total retinal layer thickness (macular thickness), nerve fibre layer (NFL), ganglion cell layer (GCL), inner plexiform layer (IPL), inner nuclear layer (INL), outer plexiform layer (OPL), outer nuclear layer (ONL), outer retinal layer (ORL) (being limited by the external limiting membrane and Bruch’s membrane, this layer corresponds to the photoreceptors) and retinal pigment epithelium (RPE).

The 6mm macular scan measurements were classified according to the ETDRS segments (‘Early Treatment Diabetic Retinopathy Study’ subfields). Central zone, inner ring, and outer ring with diameters of 1, 3, and 6 mm respectively, were included in the analysis. The average of all points within the central zone was defined as foveal thickness (1mm diameter), the inner ring (1 to 3 mm) as parafoveal thickness, and the outer ring (3 to 6 mm) as perifoveal thickness.

Neuro-ophthalmologic examination included: orthoptic status (stereopsis, cover test, motility, and clinical saccade testing). A technical saccadometry was performed using an infrared-videooculography device (see below). Saccadometry was done by a specifically trained investigator. Orthoptic examination was carried out by an experienced orthoptist.

### Video-oculographic saccadometry using EyeSeeCam in Niemann-Pick type C patients

Saccades were recorded using the infrared video-oculography (VOG) device EyeSeeCam HIT (EyeSeeTec, Fürstenfeldbruck), sampling at 220 Hz (every 3.6 ms) which was linked to a MacBook Pro 13” (OS X Version 10.9.5, Apple Inc.) equipped with the EyeSeeCam software (EyeSeeCam VOG HIT System Reversion r3429 and r3444, EyeSeeTec Fürstenfeldbruck, Germany). The study participants were seated on a height-adjustable chair, facing the center of a computer monitor (Dell Technologies Inc., 19” screen BQR-1908FPb, 1280 x 1024 resolution, 60Hz refresh rate, 300 CD/m^2^ display luminance, and 5 ms images building time) at a distance of 60 cm from the glabella to the monitor. The linear visual range was at 5°/15°/30° leftwards and rightwards and 5°/10°/20° upwards and downwards in the long-sequence saccadometry, while in the short-sequence testing only one target eccentricity was presented (20° vertically and 30° horizontally) reducing examination time. Each target was a smiley icon. Prior to the measurement, a qualitative calibration was performed. Movements of the left eye were measured.

In the healthy control cohort, saccades were counted and calculated at a threshold of 100°/s. The beginning and ending of a saccade were determined at a threshold of 2°/s. Further criteria were an occurrence 0.5 s after stimulus at latest, and an amplitude of more than 0.5 of the relevant stimuli. In the NPC cohort, this standard calculation did not detect enough saccades due to pathologically decelerated saccades. Therefore, more sensible criteria were used (threshold of 5°/s, beginning and end at 2°/s, latest detection 0.7s after stimulus and detection of saccades even if they measure less than 0.5 of the stimulus amplitude).

### Clinical severity assessment of Niemann-Pick type C

The following general clinical data were assessed: age at first symptoms and diagnosis, genotype, Filipin test, chitotriosidase activity, abbreviated 5-domain NPC Clinical Severity Scale, scale for the assessment and rating of ataxia (SARA scale), and current neurologic symptoms. The abbreviated NPC Clinical Severity Scale contains data on ambulation, manipulation, speech, swallowing and cognition. The data were obtained during routine visits in the Department of Pediatric and Adolescent Medicine.

### Statistical analysis

Regarding saccadometric measurements, age matching was not deemed necessary as the main parameters have been shown not to be age dependent in the targeted population (as described in [11]). Between the NPC group and the control group, normally distributed parameters were compared using t-tests. As this is an explorative study, we consider p-values ≤ 0.05 as indication for statistically significant difference. No adjustment for multiple testing was applied, and thus only the error rate per comparison is controlled. Spearman’s rank correlation was used to assess correlations between saccade parameters and clinical variables (NPC clinical Severity Score, SARA-Score, and Chitotriosidase activity), and between peak velocity and clinical saccadic assessment. Correlation coefficient of ≥ 0.8 was considered a strong correlation; ≥ 0.5 was considered a moderate correlation; ≥ 0.2 a weak correlation; and < 0.2 no correlation.

To analyze the differences of retinal thickness with respect to NPC disease, we used linear mixed models to control for the inclusion of one and two eyes of a study participant (as random effect). A further adjustment for age, sex, and spherical equivalent (sphere + 0.5*astigmatism) was included in the statistical analysis. Spearman’s rank correlation was conducted to correlate the outer nuclear layer (ONL) with the NPC disability score, and with horizontal peak velocity. Statistical analysis was performed using R version 4.0.0. All p-values should be regarded as continuous parameters that reflect the level of evidence from our explorative analysis and are therefore reported exactly.

## Results

A total of 18 patients (11 females, 7 males) with biochemically and/or genetically diagnosed NPC were included in the present study. A detailed clinical overview of the patient series is shown in the S1 Table. Mean age was 18 years (SD ± 9, range 6–43) in the NPC group, and 33 years (SD ± 18 years) in the control group. Most NPC patients were compound heterozygous for mutation in NPC1 gene, except two patients with homozygous mutations, and one patient, who did not exhibit a mutation in the NPC1/NPC2 gene, but revealed a classical clinical NPC phenotype in Filipin staining. All patients were on treatment with miglustat.

### Basic ophthalmologic results

Both the NPC patients and the healthy control cohort were similar regarding visual acuity, and refraction. Best corrected visual acuity was 0.0 logMAR in all NPC patients, apart from two eyes with 0.1 logMAR visual acuity, two patients with Teller Acuity Cards (binocularly tested due to reduced cooperation) logMAR 0.9 and 1.25, and one with missing data of visual acuity assessment. Refraction measurement revealed a range of spherical equivalent from -2.625 to +2.75 D with emmetropia present in 12/36 eyes, mild myopia ≤ -0.5 Diopters (D) in 19/36 eyes, mild hyperopia ≥ 0.5 D in 5/36 eyes and astigmatism ≤ -0.5 D in 15/36 eyes (minimum -1.75 D).

Anterior and posterior segments were normal in all NPC patients in the slit lamp and funduscopic examination, respectively. Optic nerve head appearance, and tortuosity grade of retinal vessels were also normal in all NPC patients.

Ocular alignment was given in all NPC patients, while stereoscopic acuity was full (40–80 arcseconds) in 9 patients and 200 arcseconds (Lang test II) in further 8 patients, while one patient presented rough stereoscopic acuity and two results were missing.

### Oculomotor investigation

During the clinical and technical assessment, we found a spectrum of compensating mechanisms to overcome vertical supranuclear saccadic gaze palsy (VSGP): We observed intensive blinking to initiate switching the gaze in nine patients, and facial spasm in one of these. Further characteristics were compensatory or defense head movements in four patients. Another technique was rightwards or leftwards trajectory (eye movements deviating towards the functioning horizontal directions) during vertical saccadic testing. Spontaneously, this compensating movement was associated with blinking (Table 1).

**Table 1:** Assessment and measurement of saccades, and OCT imaging of each NPC patient, and the respective means of the control group.

*ID*	*Age [years]*	*NPC disability score*	*Peak velocity [°/s]* *(number of recorded saccades) 30° rightwards clinical score*		*30° leftwards clinical score*		*20° upwards clinical score*		*20° downwards clinical score*		*Compensating corollary*	*Macular OCT*	*Peripapillary OCT*
1	26	8	315 (3)	1	244 (1)	1	N.A. (0)	3	N.A. (0)	3	B, H	normal	normal
2	18	11	218 (2)	0	284 (6)	0	N.A. (0)	2	N.A. (0)	1	B	normal	N.A.
3	43	11	233 (6)	0	289 (5)	0	66 (3)	3	N.A. (0)	3	B	normal	normal
4	10	9	123 (4)	0	110 (2)	0	42 (1)	2	N.A. (0)	2	B	N.A.	N.A.
5	7	5	268 (5)	0	263 (5)	0	N.A. (0)	2	359 (3)	2	B	N.A.	N.A.
6	17	2	401 (6)	0	456 (7)	0	80 (3)	2	105 (5)	2	E	normal	normal
7	28	8	318 (4)	0	383 (7)	0	112 (1)	2	53 (1)	2	B	normal	normal
8	21	12	334 (7)	0	351 (6)	0	184 (1)	2	63 (1)	2	H	normal	normal
9	14	N.A.	318 (3)	0	263 (3)	1	N.A. (0)	3	N.A. (0)	2	none	N.A.	N.A.
10	8	6	393 (4)	0	456 (2)	0	401 (4)	0	255 (4)	0	none	normal	normal
11	18	8	0		0		2		3		none	normal	normal
12	7	N.A.	0		0		2		3		none	N.A.	N.A.
13	6	N.A.	1		1		3		3		H	N.A.	N.A.
14	14	N.A.	0		0		3		1		H	N.A.	N.A.
15	21	N.A.	0		0		0		1		B	N.A.	N.A.
16	21	8	1		1		2		3		none	N.A.	N.A.
17	20	N.A.	0		0		3		2		B	N.A.	N.A.
18	19	N.A.	0		0		3		3		B	N.A.	N.A.
Controls	33 (SD 18)	N.A.	459 (SD 67) [305–677]		451 (SD 66) [259–645]		404 (SD 74) [224–609]		378 (SD 67) [216–554]		N.A.	normal	normal

B = vertical eye movements were initiated by blinking, H = compensatory or defense head movements in vertical eye movements (either upwards, or downwards, or both), E = compensating horizontal eye movements when vertical eye movements were tested.

Clinical saccade testing: 0 = normal, 1 = slowed slightly, 2 = slowed a lot, 3 = not triggerable, 4 = not assessable.

SD = standard deviation.

In the binocular clinical assessment, vertical saccades were decelerated in 16/18 patients, while one patient (ID 15) had normal saccades upwards and another patient showed clinically normal saccades in all gaze directions (ID 10). The latter one showed slightly slowed downwards saccades in video saccadometry (Table 1). Horizontal saccades were mostly normal (14/18) or slowed slightly (4/20) (Table 1). The difference between normal velocity and slightly slowed velocity was not in concordance with the measured peak velocity (no or weak correlation in horizontal saccades, see further below).

Smooth pursuit upwards and downwards was limited in eight patients, while restriction of abduction and adduction were observed in three patients.

### Saccadometric measurements

Saccadometry of NPC patients revealed the following peak velocity means and standard deviations for vertical and horizontal saccades:

rightwards 5°/15°/30°:

156°/s (SD 21°/s) (n = 5), 238° (SD 60°/s) (n = 10), 292°/s (SD 84°/s) (n = 10),

leftwards 5°/15°/30°:

173°/s (SD 43°/s) (n = 5), 285°/s (SD 88°/s) (n = 5), 310°/s (SD 105°/s) (n = 10),

upwards 5°/10°/20°:

74°/s (SD 84°/s) (n = 4), 105°/s (SD 114°/s) (n = 4), 148°/s (SD 134°/s) (n = 6),

downwards 5°/10°/20°:

84°/s (SD 68°/s) (n = 3), 90°/s (SD 50°/s) (n = 6), 167°/s (134°/s) (n = 5).

All patients underwent long- and short-sequence saccadometry. Sufficient quality data in all 12 target eccentricities in the long sequence testing was obtained in n = 3 patients, while the remainder had difficulties in performing all target eccentricities with reliable results, especially in the vertical direction. Horizontal saccades were successfully performed by all patients, at least when testing the short-sequence testing (and 5 patients also attaining reliable long-sequence testing results). Therefore, we used the data of the 30° saccades horizontally and 20 ° vertically (large target jumps) for the analysis (Table 1).

Saccade parameters, especially peak velocity, showed to be impaired in a characteristic pattern of damage: first vertically (upwards and downwards), and eventually horizontally.

**Fig 1:**
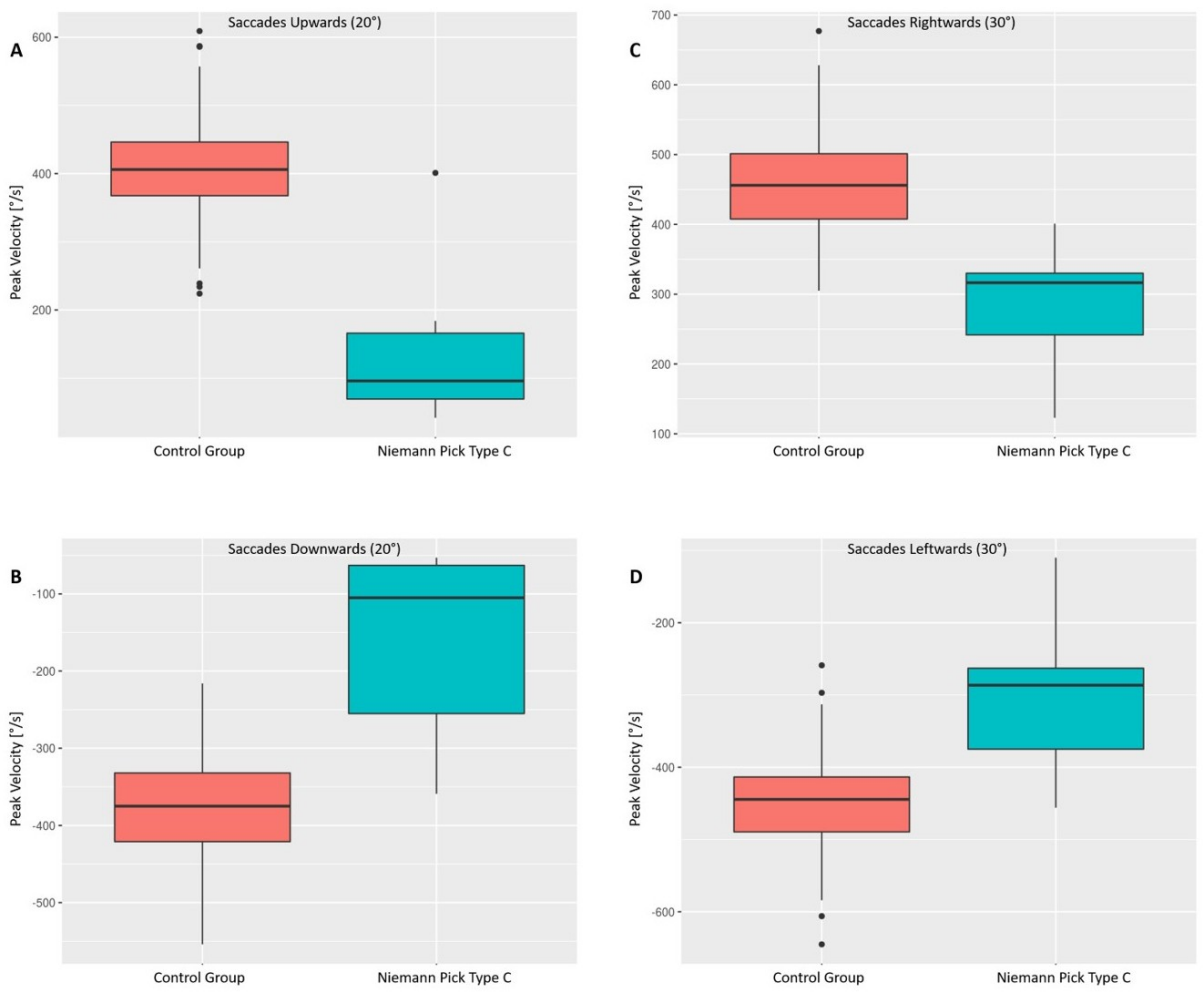
Saccades peak velocity in NPC patients is slowed compared with controls. The peak velocity (y-axis) of NPC patients vs. the control group (x-axis) is displayed for upwards (A), downwards (B), rightwards (C), and leftwards (D) saccades in boxplots. The lower boundary of the box is the 25th percentile, the line within the box indicates the 50th percentile (median) and the upper boundary represents the 75th percentile. The whiskers extend to the minimum and maximum. Outliers are displayed as dots.

In NPC patients, peak velocity was reduced to 148°/s (SD 134) upwards and 167°/s (SD 134) downwards compared to 404°/s (SD 74) upwards and 378°/s (SD 67) downwards in controls (p = 0.005 upwards and p = 0.024 downwards); see Fig 1A and 1B (20° saccades vertically).

The main sequence was preserved, as far as enough data were obtained. Likewise, horizontal saccades were slowed to 292.8°/s rightwards (controls 458.8°/s), and to 309.9°/s leftwards (controls 451.5°/s), the difference to the control group was statistically significant (p = 0.0001 and p = 0.002 respectively). The data are provided as boxplots in Fig 1C and 1D (30° saccades horizontally).

We found strong negative correlations between horizontal peak velocity and SARA-Score (rho = -0.81, p = 0.05), and between vertical gain and NPC Clinical Severity Score (rho = -0.94, p = 0.02). No significant correlation was found with chitotriosidase activity. Peak velocity did not significantly correlate with clinical saccadic assessment (upwards: rho = -0.68, p = 0.14; downwards: rho = -0.35, p = 0.56; leftwards: rho = -0.48, p = 0.16; rightwards: rho = -0.06, p = 0.87).

### Foveal retinal thinning in macular OCT

The macular OCT analysis revealed significant thinning of the total retinal thickness in the fovea, and specifically of the foveal outer nuclear layer (ONL) and outer retinal layer (ORL), forming the main layers in the fovea. These findings remained significant after adjusting for age, sex, and spherical equivalent. The complete analysis of all retinal layers is shown in Table 2. No correlation was found between the foveal ONL and the NPC-disability score (rho = -0.06, p = 0.25), the genotype (0 homozygous, 1 heterozygous) (rho = -0.36, p = 0.25) and horizontal peak velocity of reflexive saccades (rho = 0.3, p = 0.25). Peripapillary RNFL thickness was not different in NPC compared to the control cohort (S2 Table shows the global and sectorial mean values).

**Table 2:** Distribution of retinal layer measurements from SD-OCT in NPC and controls. A linear mixed model was used for statistical analysis. In the adjusted model, age, sex, and spherical equivalent were included.

*Macular OCT layers and ETDRS segments*	*Control eyes (n = 180) Mean (SD) [μm]*	*NPC eyes (n = 14) Mean (SD) [μm]*	*Crude model p-value*	*Adjusted model p-value*
1) NFL				
Fovea	12.7 (2.1)	11.9 (2.1)	0.30	0.22
Inner Ring	21.7 (1.8)	21.1 (1.5)	0.50	0.44
Outer Ring	36.3 (4.0)	37.7 (5.6)	0.43	0.68
2) GCL				
Fovea	16.1 (4.4)	14.7 (3.8)	0.35	0.12
Inner Ring	52.3 (4.2)	52.6 (4.0)	0.93	0.59
Outer Ring	35.9 (3.1)	37.8 (2.8)	0.31	0.49
3) IPL				
Fovea	22.0 (3.7)	20.8 (2.8)	0.34	0.15
Inner Ring	42.7 (2.8)	42.8 (2.8)	0.88	0.71
Outer Ring	29.5 (2.5)	31.0 (2.1)	0.21	0.31
4) INL				
Fovea	18.7 (4.6)	15.9 (3.9)	0.08	0.09
Inner Ring	40.3 (2.7)	39.7 (3.6)	0.58	0.44
Outer Ring	33.3 (2.4)	34.7 (2.3)	0.34	0.74
5) OPL				
Fovea	26.2 (5.3)	25.2 (5.0)	0.59	0.37
Inner Ring	33.5 (3.8)	33.4 (3.8)	0.98	0.80
Outer Ring	27.1 (1.7)	27.5 (1.8)	0.50	0.39
**6) ONL**				
Fovea	**92.5 (10.3)**	**80.4 (8.5)**	**0.002**	**0.007**
Inner Ring	72.2 (8.6)	65.8 (5.5)	0.06	0.10
Outer Ring	59.8 (6.5)	55.9 (4.6)	0.12	0.12
**7) ORL (ELM to BM)**				
Fovea	**90.6 (4.1)**	**87.6 (4.6)**	**0.027**	**0.010**
Inner Ring	81.6 (2.3)	80.3 (2.2)	0.13	0.12
Outer Ring	78.3 (2.1)	78.3 (2.0)	0.96	0.98
7) RPE				
Fovea	17.1 (1.8)	16.9 (1.9)	0.88	0.74
Inner Ring	14.8 (1.5)	14.0 (1.6)	0.15	0.38
Outer Ring	13.1 (1.2)	12.9 (1.3)	0.97	0.64
**Total retinal thickness**				
Fovea	**278.3 (19.2)**	**256.1 (21.1**)	**0.003**	**0.001**
Inner Ring	343.8 (12.8)	334.9 (15.3)	0.12	0.07
Outer Ring	306.9 (20.7)	306.3 (17.2)	0.79	0.60

SD-OCT = spectral domain optical coherence tomography, NPC = Niemann-Pick type C, ETDRS segments: OCT division scheme being named after the ‘Early Treatment Diabetic Retinopathy Study’, SD = standard deviation, NFL = nerve fibre layer, GCL = ganglion cell layer, IPL = inner plexiform layer, INL = inner nuclear layer, OPL = outer plexiform layer, ONL = outer nuclear layer, ORL = outer retinal layer (ELM = external limiting membrane, BM = Bruch’s membrane), RPE = retinal pigment epithelium, (I)1 = (inferior) inner ring, (I)2 = (inferior) outer ring.

## Discussion

We report ocular findings in a case series of 18 NPC patients, characteristic saccadometric results in a subgroup of 10, and OCT measurements in another subgroup of 8 patients. In the complete cohort, there were no anatomical abnormalities in the anterior segments, which is in line with previous data (S3 Table), with only one case reported to exhibit iridocyclitis [12].

Foveal retinal thinning, specifically in the foveal ONL and ORL (photoreceptors), was detected by OCT imaging. This finding is in line with animal data: Claudepierre et al. showed in the NPC mice models the ONL histologically to exhibit conspicuous folds, and displaced nuclei lined up along the ONL folds in the OPL (which itself showed focal thinning). Strongly deformed and degenerated photoreceptor outer segments were present, which theoretically might correlate to our thinned ORL [13].

To the contrary we cannot support findings by Havla et al., who reported reduced volumes of macular NFL, as well as a thinner peripapillary RNFL, and combined GCL/IPL, compared to age- and sex-matched controls in their study. Of note, their patients exhibited reduced Snellen Visual Acuity Equivalent, while in our cohort almost all patients had normal visual acuity [14].

The involvement of saccadic eye movements in NPC ranges from no impairment as seen in one patient in the present study and as reported by Greenberg et al. [15], to the full extent of VSGP. Furthermore, even asymptomatic first-degree relatives of NPC patients, demonstrate oculomotor abnormalities [16]. Specific saccadic parameters have already been used to assess disease progression and treatment success of miglustat in NPC [2, 6]. Peak velocity is one important quantitative functional parameter, which is slowed vertically as a result of VSGP [17]. Similar to Lengyel et al. [18], we were not able to confirm downwards accentuated paralysis, as found in other studies [9, 19]. The spread from vertical to horizontal saccadic dysfunction or saccadic gaze paralysis in the course of the disease is indicated in other studies [18, 20, 21]. We detected horizontal involvement in 4/18 patients, compared to 1/11 in a study by Chamova et al. [22]. The NPC-registry indicates 4.9% of horizontal gaze palsy. As the disease progresses, impairment of horizontal eye movements can be identified, and vertical saccades can be almost absent [21]. In many cases, vertical saccadic paralysis is severe, impeding recording and measuring vertical saccade parameters [23]. In the present cohort, only one half of those who performed VOG, produced fairly reliable VOG measurements in vertical saccades, while this is already a selected, cooperating patient population. Unlike saccadometry in Gaucher patients [24], it is often quite difficult to examine severely affected NPC patients. However, saccadometry might help to more accurately evaluate and quantify unidentified or clinically subnormal saccades in NPC patients over time. Particularly regarding the parameter gain, a strong correlation with specific brain volumetry was found in the past [8]. Peak velocity and gain showed to correlate with clinical parameters in our small study sample. Saccadic parameters could demonstrate progression of NPC, and possibly also the effectiveness of new therapies in the future, as it is an expression of CNS involvement. Clinical orthoptic examination remains essential, because accompanying symptoms such as blinking [25] and compensatory head movements [26] are mainly captured clinically, and even interfere with saccade measurement. Most common feature to compensate VSGP was intensive blinking with eventual facial spasm to initiate switching the gaze, which was also reported by Shawkat et al. [25]. Short and repeated eye movements (involuntary jerky movements) towards the functioning horizontal directions during vertical saccadic testing as termed ‘round the houses’ [27] was noted in a comparable pattern in one of our study patients. These signs have been reported in Parkinson’s disease as well [28].

Despite the low prevalence of NPC of 0.66–0.83/100,000 in Europe [1] we were able to investigate a series of 18 NPC patients, and to perform comprehensive video saccadometry, and OCT imaging in subgroups. Due to the small sample size encountered in this ultra-rare disease, and the small amount of data, which is mainly a result of difficult compliance during the examination, only the parameter peak velocity could be evaluated in detail, while other parameters such as gain were not of reliable quality. There is a possible selection bias, because patients who are able to undergo such an extensive examination typically present a mild or intermediate disease severity. As NPC is a progressive disease, long-term OCT data would be of particular interest to detect retinal degeneration in the individual patient.

To summarize, the pattern of saccadic impairment in NPC affects predominantly vertical, and eventually horizontal eye movements. Characteristic compensatory mechanisms of VSGP during vertical eye movement testing include blinking, head movements and horizontal trajectory. These can interfere with saccade measurements in such a way that only moderately affected patients may be measured, or that only the horizontal saccades may be measured.

Saccadometry, in contrast to clinical investigation, allows the precise evaluation of saccades in NPC patients. Saccadic impairment is an expression of CNS involvement and therefore a parameter to demonstrate the progression of NPC, and possibly also the efficacy of new therapies. Though it seems reasonable to assume that foveal thinning may be indicative of central nervous involvement (as in other neurodegenerative disorders), this still needs confirmation in studies specifically addressing this issue. Apart from these neuro-ophthalmologic aspects in NPC patients supporting existing literature, foveal retinal thinning specifically of the ONL and ORL (photoreceptors) was demonstrated in patients with an otherwise regular ophthalmologic examination. Thus, in a next step, the retinal diagnostic in NPC should be investigated in more detail, as the natural course of these changes is not yet understood. In consequence, it remains to be shown, whether OCT will prove useful to monitor progression in the future.

## Supporting information

S1 Table Clinical data of NPC patients. *null mutation.
(PDF)

S2 Table Peripapillary retinal nerve fibre layer thickness in NPC eyes and controls. RNFL = retinal nerve fibre layer, SD = standard deviation.
(PDF)

S3 Table Reported ocular abnormalities in Niemann-Pick disease type C. (PDF)
