# Supplementary material for: Vertical saccadic palsy and foveal retinal thinning in Niemann-Pick disease type C
Source: PLoS One. 2021 Jun 4;16(6):e0252825. doi: 10.1371/journal.pone.0252825 (PMC8177533; doi:10.1371/journal.pone.0252825)
Supplement: S1 Table — *null mutation. (PDF) [file pone.0252825.s001.pdf]

S1 Table. Clinical data of NPC patients.

ID	*Age*	*Age of specific symptoms / at diagnosis*	*First symptom(s)*	*Mutation analysis*	*Filipin test (evaluation of the test results by the lab)*	*Chitotriosidase activity (normal value 20-100) (nmol/ml/h)*	*NPC Clinical Severity Score*	*SARA-Score*	*Neurologic symptoms*
1	26	21	jaundice, restlessness, stuttering, loss of educational skills, saccades	compound heterozygosity NPC1 gene [p.Y825C/p.A1054T]	classical NPC phenotype	349	8	13	cognitive impairment, ataxia, dystonia, dysarthrophonia bilateral hearing loss
2	18	10	hepatosplenomegaly, impaired motor development, neurologic abnormalities	compound heterozygosity NPC1 gene [p.S940L/p.P1007A]	N.A.	1492	11	26	cognitive impairment, ataxia, cataplexy, epilepsy
3	43	34	progressive cognitive impairment, coordination difficulties, lack of motivation, depressive mood	compound heterozygosity NPC1 gene [p.P434L/p.I1061T]	clearly positive	3200	11	N.A.	dementia, ataxia, dystonia, dysarthria, personality disorder, depression
4	10	7	delayed motor development, dysarthria	compound heterozygosity NPC1 gene [p.A97V/p.S1004P]	N.A.	203	9	N.A.	cognitive impairment, ataxia, dysarthria, cataplexy, epilepsy
5	7	4	impaired speech development	compound heterozygosity NPC1 gene [p.A97V/p.S1004P]	N.A.	N.A.	5	N.A.	cognitive impairment, ataxia, dysarthria, dysphagia, cataplexy
6	17	10	dysarthria	homozygous NPC1 gene [p.G1162 A/p.G1162 A]	massively positive	1*	2	3	cognitive impairment, dysmetria
7	28	21	forgetfulness, psychosis	homozygous NPC1 gene [p.G1162A/p.G1162A]	N.A.	338	8	13	cognitive impairment, ataxia, dysarthria, dysphagia
8	21	14	loss of educational skills, motor and coordination disorder	compound heterozygosity NPC1 gene [p.S954L/p.IVS23+1G>A]	significantly positive	116	12	15	cognitive impairment, ataxia, dystonia, dysarthria, dysmetria, dysdiadochokinesia, epilepsy, bilateral hearing loss
9	14	11	seizures	compound heterozygosity NPC1 gene [p.IVS1+2T>c/p.P1007A]	positive	418	N.A.	N.A.	cognitive impairment, ataxia, dysarthria, epilepsy, dystonia
10	8	8	enuresis	compound heterozygosity NPC1 gene [p.S954L/p.S849I]	N.A.	244	4	0	ataxia, bilateral hearing loss
11	18	12	cognitive impairment	no mutation detected in NPC1+2 gene (null mutation)	classical NPC phenotype	38	8	7	cognitive impairment, ataxia, dysmetria, dysarthria
12	7	5	loss of cognitive and motor skills	compound heterozygosity NPC1 gene [p.fs1005*/p.P1007A]	N.A.	279	N.A.	N.A.	development delay, ataxia
13	6	4	feeding difficulties, developmental delay	compound heterozygosity NPC1 gene [p.1061T/p.Q447Tfs*12]	massively positive	699	N.A.	N.A.	cognitive impairment, ataxia, dysmetria, muscular hypotonia
14	14	8	hepatosplenomegaly	compound heterozygosity NPC1 gene [p.M1001V/p.R404W]		323	N.A.	N.A.	cognitive impairment, cataplexy, dysarthria, dystonia, epilepsy
15	21	17	developmental delay	heterozygosity NPC1 gene [p.P237S/-]	significantly increased	63	N.A.	12	mental retardation, ataxia, dysarthria, dysphagia, dysmetria
16	21	Child-hood	stuttering, disturbed eye movements	compound heterozygosity NPC1 gene [p.I1061T /p.P1007A]	N.A.	N.A.	8	7	cognitive impairment, ataxia, dysarthria, dysphagia,
17	20	20	learning disability, gross and fine motor weakness	compound heterozygosity NPC1 gene [p.I1061T/p.A1151T]	N.A.	160	N.A.	11	cognitive impairment, Ataxia, dysmetria, dysdiadochokinesia, dysarthria, epilepsy
18	19	4	protruding abdomen, thrombopenia	compound heterozygosity NPC1 gene [p.I1061T/p.E1188X]	N.A.	736	N.A.	16	Ataxia, dysarthria, cataplexy, dysphagia, dystonia, epilepsy

*null mutation
