# Supplementary material for: Vertical saccadic palsy and foveal retinal thinning in Niemann-Pick disease type C
Source: PLoS One. 2021 Jun 4;16(6):e0252825. doi: 10.1371/journal.pone.0252825 (PMC8177533; doi:10.1371/journal.pone.0252825)
Supplement: S2 Table — RNFL = retinal nerve fibre layer, SD = standard deviation. (PDF) [file pone.0252825.s002.pdf]

S2 Table. Peripapillary retinal nerve fibre layer thickness in NPC eyes and controls.

Peripapillary RNFL Mean (SD) [µm]	*Control eyes (n= 178)*	*NPC eyes (n=14)*	*p-value*
Global (average)	96.7 (9.8)	99.9 (8.1)	0.37 (0.28)
Nasal-inferior	105.8 (23.6)	103.2 (19.5)	0.76 (0.70)
Nasal	73.6 (15.6)	77.9 (10.7)	0.44 (0.22)
Nasal-superior	106.3 (19.8)	103.6 (11.5)	0.71 (0.93)
Temoral-superior	135.0 (18.0)	140.2 (14.6)	0.41 (0.48)
Temporal	69.9 (10.5)	73.4 (8.8)	0.35 (0.58)
Temporal-inferior	139.3 (19.3)	150.1 (20.4)	0.1 (0.11)

RNFL = retinal nerve fibre layer, SD = standard deviation.
