# Supplementary material for: Vertical saccadic palsy and foveal retinal thinning in Niemann-Pick disease type C
Source: PLoS One. 2021 Jun 4;16(6):e0252825. doi: 10.1371/journal.pone.0252825 (PMC8177533; doi:10.1371/journal.pone.0252825)
Supplement: S3 Table — (PDF) [file pone.0252825.s003.pdf]

S3 Table. Reported ocular abnormalities in Niemann-Pick disease type C.

Structure	*Number of patients studied*	*Ocular abnormality*			*Age (years)*	*References*
Conjunctiva	1	Abnormal ultrastructure (lamellar cytoplasmic bodies)			5	Merin et al., 1980 [15]
Anterior segments	1	Heterochromic iridocyclitis and vertical motility limitation			26	Kianersi and Sonbolestan, 2017 [12]
Macula + optic disc	14	Spectral-domain OCT in NPC patients, mutation carriers and controls: lower volumes of macular retinal nerve fibre layer, and lower combined ganglion cell and inner plexiform layer in patients compared to controls (not in mutation carriers).			9-38	Havla et al. 2020 [14]
Retina	-	NPC1 2-months-old mice: Impaired visual function, accumulation of lipofuscin in the retinal pigment epithelium layer, degeneration of photoreceptor outer segments, disruption of synaptic layers, increased autophagy markers in the ganglion cell layer, folds of the outer nuclear layer			-	Claudepierre et al., 2010 [13]
<u>Oculomotor system</u>		<u>Vertical gaze palsy (saccadic or pursuit palsy)</u>	<u>Horizontal gaze palsy</u>	<u>Vestibulo-ocular reflex</u>		
	2	X	X	Normal	27, 34	Lengyl et al., 1999 [16]
	1	X downwards, subsequently upwards	Normal	Normal	8	Shawkat et al., 1994 [17]
	- (Review)	X	Relatively spared	X	-	Salsano et al., 2012 [9]
	9	NA	X hypometria, and slightly reduced saccadic peak velocity	Normal	18-49	Abel et al., 2012 [18]
	9	NA	X brain volumes correlate with saccadic gain	NA	18-49	Abel & Walterfang et al., 2012 [7,8,18]
	1	Normal (asymptomatic: without VSGP, without neurological impairment)	NA	Normal	66	Greenberg et al., 2015 [19]
	472	X 68%	X 4.9%	NA	21 ± 15	Patterson et al., 2015+2020 [3,5]
	11	X all patients	X limitation of horizontal eye movements (n=1)	NA	14 ± 8	Chamova et al., 2016 [20]
	1	X 'Round the houses' sign	NA	NA	59	Eggink et al., 2016 [21]
	1	X saccades, optokinetic nystagmus, and smooth pursuit affected	NA	NA	1	Bremova and Strupp, 2017 [22]
	2+3	X slower vertical peak velocity and greater curvature compared to 77 controls	Almost entirely unaffected	NA	2-9	Blundell et al., 2018 [23]
	1	X Impaired smooth pursuit, saccades and optokinetic nystagmus (more prominent in downward direction)	Relatively preserved	NA	30	Gupta et al., 2018 [24]
	20 First-degree heterozygous relatives of patients with NPC	X shortened duration of reflexive saccades, prolonged duration of self-paced saccades, elevated gaze-holding nystagmus on up gaze	X reduced horizontal peak velocity of self-paced saccades	Normal in horizontal plane	53 ± 10	Bremova et al. 2020 [29]

X= yes / abnormal
